# Integrating computational modeling, neuroimaging, and neuromodulation to decompose perceptual decision-making in healthy aging: a review of methods, findings, and gaps

**DOI:** 10.3389/fnagi.2026.1906284

**Published:** 2026-08-07

**Authors:** Anna Udoratina, Vladimir Maksimenko, Susanna Gordleeva

**Affiliations:** 1 Institute of Neuroscience, National Research Lobachevsky State University of Nizhny Novgorod, Nizhny Novgorod, Russia; 2 Neuromorphic Computing Center, Neimark University, Nizhny Novgorod, Russia; 3 Innopolis University, Innopolis, Russia; 4 Department of Neurotechnology, Lobachevsky University, Nizhny Novgorod, Russia

**Keywords:** drift diffusion model, healthy aging, non-invasive brain stimulation, perceptual decision-making, sequential sampling models

## Abstract

Perceptual decision-making (PDM) — the transformation of sensory input into behavioral choice — declines with healthy aging, leading to slower responses, altered accuracy, and negative impacts on quality of life. Recent advances in model-based cognitive neuroscience, particularly drift-diffusion models (DDMs), allow decomposition of PDM into latent components (e.g., evidence accumulation, decision caution, non-decision time) and enable identification of neural circuits associated with each component. This decomposition provides a foundation for targeted neuromodulatory interventions (e.g., transcranial magnetic stimulation) aimed at mitigating age-related cognitive decline. Although a substantial body of literature has successfully combined DDM with neuromodulation to enhance distinct PDM components in young adults, evidence in older populations remains critically limited. Due to age-related morphological, physiological, and connectivity changes, findings from young adults cannot be directly generalized to older adults. This review highlights this research gap and calls for systematic investigation at the intersection of cognitive modeling and neuromodulation to selectively improve distinct components of perceptual decision-making in aging.

## 1 Introduction

Perceptual decision-making (PDM) is one of the most fundamental processes affected by aging. It refers to the ability to extract sensory information from the environment and transform it into categorical judgments, thereby supporting a wide range of everyday behaviors, such as recognizing faces in a crowd or estimating the speed of an approaching vehicle. A substantial body of behavioral research demonstrates that healthy aging is associated with a decline in PDM performance, typically reflected in slower reaction times (RTs) and, in some cases, reduced accuracy (e.g., Bolam et al., 2024; Forstmann et al., 2011; Ratcliff et al., 2007, 2006).

For decades, the dominant account of age-related RT increase has been the general slowing hypothesis (Salthouse, 1996), which posits a uniform decline in processing speed across all cognitive stages. However, RTs being the aggregate outcome do not permit discrimination between different cognitive processes that are involved in PDM. Specifically, it remains unclear whether aging primarily affects the quality of sensory evidence accumulation, increases the conservatism of the decision criterion, prolongs motor execution, or reflects a combination of these factors. This limitation has motivated the adoption of mathematical models that conceptualize PDM as the interaction of multiple latent subprocesses, among which the drift-diffusion model (DDM) has received particular attention in this field (Forstmann et al., 2016). The DDM provides a principled framework for decomposing PDM into latent components, including drift rate (reflecting the efficiency of evidence accumulation), decision threshold (capturing response caution), and non-decision time (encompassing sensory encoding and motor execution) (Ratcliff, 1978; Ratcliff et al., 2016).

While an extensive body of literature has demonstrated the promise of applying the DDM framework to gain insights into aging, the relationship between age-related decline in specific cognitive components and the activity of brain regions remains an important open question. From a fundamental perspective, addressing this issue would deepen our understanding of the interplay between cognitive and neurobiological aspects in the aging process. From a practical perspective, it could enable targeted therapeutic interventions through direct modulation of relevant regions using non-invasive neurostimulation.

Alongside their potential therapeutic effects, neuromodulation techniques provide a valuable tool for testing hypotheses about the causal relationships between activity in specific brain regions and declines in distinct cognitive components of perceptual decision-making (PDM). At the same time, although substantial progress has been made in establishing these causal relationships, the strength of the reported causal evidence varies considerably across studies, depending on the neuromodulation technique employed (e.g., transcranial electrical stimulation, tES, or transcranial magnetic stimulation, TMS), the stimulation protocol, and the experimental design.

Our literature review shows that the vast majority of studies investigating the neurobiological basis of PDM and its neuromodulation have been conducted on samples of young adults. Although this body of work provides evidence for causal relationships between activity in specific brain regions and different components of PDM, as well as the potential to improve these cognitive components through stimulation, direct extrapolation of these findings to older adults is methodologically unjustified. Brain aging is accompanied by fundamental changes, including the degradation of gray and white matter (Groh and Simons, 2025; Lockhart and DeCarli, 2014), alterations in neurotransmitter system dynamics (Mora et al., 2007), decreased sensory sensitivity (Li and Lindenberger, 2002), and changes in the hemodynamic response (Zimmerman et al., 2021). Together, these factors create a qualitatively different neurobiological environment in which the same neural circuits may function differently, and the effects of the brain stimulation may be less predictable.

The aim of this review is to synthesize evidence from the past two decades on age-related changes in PDM, with a particular emphasis on integrative approaches combining computational modeling, neuroimaging, and neuromodulation. The review is structured as follows. First, we outline the physiological changes associated with aging that affect brain structure and functions. Second, we summarize behavioral findings and insights derived from sequential sampling models. Third, we examine neuroimaging studies that have identified the neural correlates of PDM. Fourth, we review studies that combine non-invasive brain stimulation with computational modeling to establish causal links between brain regions and specific components of the decision process, highlighting the scarcity of such work in older adults. Finally, we identify key gaps in the literature and propose directions for future research.

Based on the review results, we argue that the systematic integration of modeling, neuroimaging, and neuromodulation in aging samples is scarce and urgently needed. Such research would not only test the generalizability of existing cognitive models from young to older populations but also inform the development of targeted interventions to mitigate age-related cognitive decline. We note that this review is not exhaustive for a broader perspective on the topics discussed, readers may refer to the following works (Dully et al., 2018; Heekeren et al., 2008; Mulder et al., 2014).

## 2 Methodology

In this review, we focused exclusively on work related to healthy aging, so studies examining PDM in the context of pathological conditions were excluded. Searches were conducted in Google Scholar and PubMed. Inclusion criteria were: (1) relevance to the keywords “perceptual decision-making,” “older adults,” “sequential sampling model,” “DDM,” “fMRI,” “NIBS,” “TMS,” “tES”; (2) peer-reviewed articles; (3) publication period from 2010 to 2025; (4) studies involving conditionally healthy older and young adults.

We focus only on visual tasks since the visual system is the most studied model of PDM, providing a high degree of methodological standardization across studies. Moreover, excluding auditory, tactile, and multimodal tasks minimizes heterogeneity associated with different rates of sensory aging, thus isolating age-related variance in the decision process from modality-specific sensory deterioration. Although studies employ tasks across different sensory modalities, such as tactile (Hegner et al., 2015, 2017; Preuschhof et al., 2006) or auditory stimuli (Amitay et al., 2013; Binder et al., 2004; Kaiser et al., 2007; Mulert et al., 2008), but visual paradigms are the most common (for a more detailed review of PDM in various task modalities, see Heekeren et al., 2008).

For sections reviewing age-related differences in behavioral data and SSM parameters, additional criteria required that the study directly compared young and older adults in terms of mean RT, accuracy, and SSM parameter estimates. For both the neuroimaging and NIBS sections, we include studies that employ an SSM either for behavioral data analysis or as a theoretical framework for interpreting fMRI data. Brief description of the PDM tasks used in the reviewed studies is given in Supplementary Table 1.

## 3 Age-induced physiological changes in the brain

Age-related decline in cognitive test performance is associated with structural, physiological, and neurochemical changes (Park and Reuter-Lorenz, 2009). Cognitive decline in older adults is primarily attributed to gray and white matter atrophy (Armstrong et al., 2020; Burzynska et al., 2010; Raz and Rodrigue, 2006; Resnick et al., 2003; Revie and Metzler-Baddeley, 2024), beginning at approximately age 50 (Sexton et al., 2014). This includes cortical thinning in the fronto-parietal region, which is considered a key regulator of activity in other areas (Burzynska et al., 2010; Cole et al., 2013; Rieck et al., 2017), and reduction in white matter integrity, which is also strongly associated with declines in executive function efficiency and information processing speed (Bennett and Madden, 2014; Petkoski et al., 2023). In this context, Rasooli et al. (2021) also reported reduced structural connectivity between the prefrontal cortex and striatum, which partially mediated age-related slowing of action selection. Similarly, Coxon et al. (2012) found that the integrity of preSMA–subthalamic nucleus projections declines with age and predicts individual differences in inhibitory control (stop-signal reaction time), with the strongest effects observed in older adults.

At the metabolic level, aging is associated with several processes that negatively affect nervous system function. These include mitochondrial dysfunction (DiMauro and Schon, 2008), leading to impaired energy metabolism; oxidative stress (Sims-Robinson et al., 2013), causing damage to cellular structures; and disrupted autophagy processes (Palmer et al., 2025). These factors collectively compromise neuronal integrity and the blood–brain barrier, whose condition correlates with the preservation of cognitive function (Erickson and Banks, 2019).

At the neurochemical level, aging is characterized by altered neuromodulation and neurotransmitter balance. Particular importance in the context of cognitive aging is attached to the age-induced alteration of dopaminergic system (Berry et al., 2019). Reduced dopamine receptor density is associated with increased intra-individual behavioral variability (Karalija et al., 2024; MacDonald et al., 2012); and dopamine synthesis levels are related to task-switching efficiency, but the nature of this relationship differs by age (Berry et al., 2016, 2018). In younger adults, higher dopamine directly facilitates the neural connectivity that supports flexible performance. In older adults, this dopaminergic facilitation is disrupted; however, those with sufficiently elevated dopamine synthesis still demonstrate the beneficial link between connectivity and task-switching efficiency (Berry et al., 2018). The integrity of the cholinergic system has also been noted as crucial for maintaining cognitive function, with age-related decline in its structural integrity associated with lower performance in attention and episodic memory (Grothe et al., 2013; Nemy et al., 2020).

Age-induced neurobiological changes translate into declines in sensory, motor, and cognitive functions that are essential for PDM. Declining performance in PDM tasks, may be partly attributable to impaired sensorimotor system function (Gómez-Granados et al., 2021). Firstly, aging is associated with visual impairments (Hutchinson et al., 2012; Owsley, 2011; Spear, 1993), hearing loss (Goossens et al., 2017; Slade et al., 2020), and reduced tactile sensitivity (Löffler et al., 2024; Samain-Aupic et al., 2024). According to the sensory load concept, weakening of sensory systems leads to increased cognitive load, placing higher demands on the resources involved in PDM (Monge and Madden, 2016) and contributing to the development of cognitive deficits (Fischer et al., 2016; Maharani et al., 2018). Age-related loss of sensory sensitivity results in noisier perceptual representations (Dully et al., 2018). As for motor functions, normal aging is associated with loss of motor neurons and reduced corticospinal tract integrity. These changes result in increased motor output variability, reduced fine motor control, and prolonged movement execution times (Gordleeva et al., 2026; Hunter et al., 2016).

Beyond sensory and motor decline, aging affects higher-order cognitive functions that are essential for PDM such as executive functioning, attention and memory (Harada et al., 2013). Cognitive control — the ability to maintain task goals, inhibit irrelevant information, and update working memory — shows reliable age-related decline (Braver, 2012), which is linked to reduced prefrontal recruitment and weaker default mode network suppression (Turner and Spreng, 2015). Working memory capacity, in particular, is reduced in older adults, and this reduction correlates with declines in fronto-parietal integrity and reduced dopamine levels (Nyberg et al., 2012). Age-related impairments in executive control are also reflected in a reduced ability to flexibly adjust decision thresholds under changing speed and accuracy demands (Forstmann et al., 2011). Because PDM often requires maintaining task instructions, integrating sensory evidence over time, and resisting premature responses, declines in cognitive control, attention, and working memory directly affect decision quality and speed.

The multifaceted neural alterations described above may contribute to declines in performance on PDM tasks in several ways. Sensory decline may lead to noisier perceptual representations, increasing the amount of evidence required to reach a decision. Reduced white matter integrity may slow the transmission of evidence from sensory to decision and motor regions. Impaired cognitive control and reduced flexibility in adjusting decision thresholds may cause older adults to persist with suboptimal decision strategies. Therefore, it is important to examine the effects of aging on different components of PDM and identify the brain regions whose activity is associated with the decline of these components.

## 4 Age-related changes in the perceptual decision-making process effectiveness

The primary behavioral marker of age-related changes in PDM is increased RTs. RT is usually higher in older adults regardless of the tasks and experimental paradigms used (Hultsch et al., 2002). Among the studies reviewed in this article and summarized in Supplementary Table 2, the only exception was the gradual contrast-change detection task (McGovern et al., 2018), where no differences in RT were observed. The increase in RT with age may be explained by functional and structural changes in the brain. First, age-related changes in RT may be associated with deterioration of brain functions that are closely related to the performance in PDM task, including executive control functions (Ferguson et al., 2021), attention (Wasylyshyn et al., 2011) [although some aspects may remain stable or even improve with age as demonstrated in Veríssimo et al. (2022)], working memory (Reinhart and Nguyen, 2019; Vaqué-Alcázar et al., 2020), and sensory system (Fischer et al., 2016; Owsley, 2011; Roberts and Allen, 2016). Second, RT may increase with age due to structural disruptions in the central nervous system (Kuznetsova et al., 2016) including changes in white matter, weakening cortico-cortical and subcortical-cortical connections (Hardwick et al., 2022; Levin et al., 2011).

Along with RT, accuracy is an important indicator of the PDM process. Unlike the relatively consistent findings for RT, results regarding age-related changes in accuracy are more variable. Some studies report no significant age-related differences in accuracy between younger and older adults (Ratcliff et al., 2007; Revie and Metzler-Baddeley, 2024; Kavcic et al., 2013). Other studies highlight that age-related differences in accuracy may emerge for specific stimuli and instructions. For instance, in the brightness discrimination task, younger adults showed significantly higher accuracy only under accuracy-focused instructions (Ratcliff et al., 2006). In the letter discrimination task, younger adults demonstrated better accuracy only for stimuli with high spatial frequency (Ratcliff et al., 2007). Finally, in those papers where age related differences exist, authors usually report their relatively small magnitude, suggesting that older adults perform the tasks almost as successfully as younger adults.

Taken together, the reviewed literature consistently demonstrates that healthy aging is strongly associated with increased RTs in PDM tasks, and this slowing is observed across a wide range of stimuli and task instructions. In contrast, the pattern for decision accuracy is markedly different: age-related differences are inconsistent, highly task-specific, and sometimes absent or low in magnitude.

## 5 Perceptual decision-making and sequential sampling models

Perceptual decision-making is defined as the process of choosing between alternatives based on the processing of noisy sensory information. The theoretical framework of PDM is often formalized using a wide class of mathematical models known as sequential sampling models (SSMs). SSMs assume that during decision-making, an observer accumulates noisy samples of sensory information until a threshold is reached (for review, see Forstmann et al., 2016). The close connection between the SSMs and neurophysiology has been a major factor driving the model’s widespread adoption.

According Shadlen and Kiani (2013), detection of a sensory stimulus in the perceptual field is associated with increased neuronal activity in sensory areas. Next, a decision variable is formed, which the authors describe as the difference in spike counts between neurons representing alternative response options. The evidence accumulation stage involves the temporal integration of this difference until it reaches a certain threshold — that is, a stereotyped level of firing rate in decision-related neurons. The moment the threshold is reached corresponds to the completion of the decision process. Finally, the cycle ends with the planning and execution of a motor response. Practically, in monkeys performing motion discrimination tasks, single-unit recordings have shown that neurons in the lateral intraparietal area (LIP) display a gradual increase in firing rate that closely tracks the accumulation of sensory evidence (Gold and Shadlen, 2007; Roitman and Shadlen, 2002). Furthermore, electrical stimulation of LIP biases perceptual choices, offering causal evidence that this region is involved in forming decisions (Hanks et al., 2006).

Among SSMs (for review, see Ratcliff et al., 2016), the DDM (Ratcliff, 1978) has emerged as the most widely validated framework for analyzing perceptual decisions. The DDM’s prominence stems from several key features. First, it simultaneously accounts for three types of behavioral data: the proportion of correct responses, the distribution of RTs for correct responses, and the distribution of RTs for errors.

**FIGURE 1:**
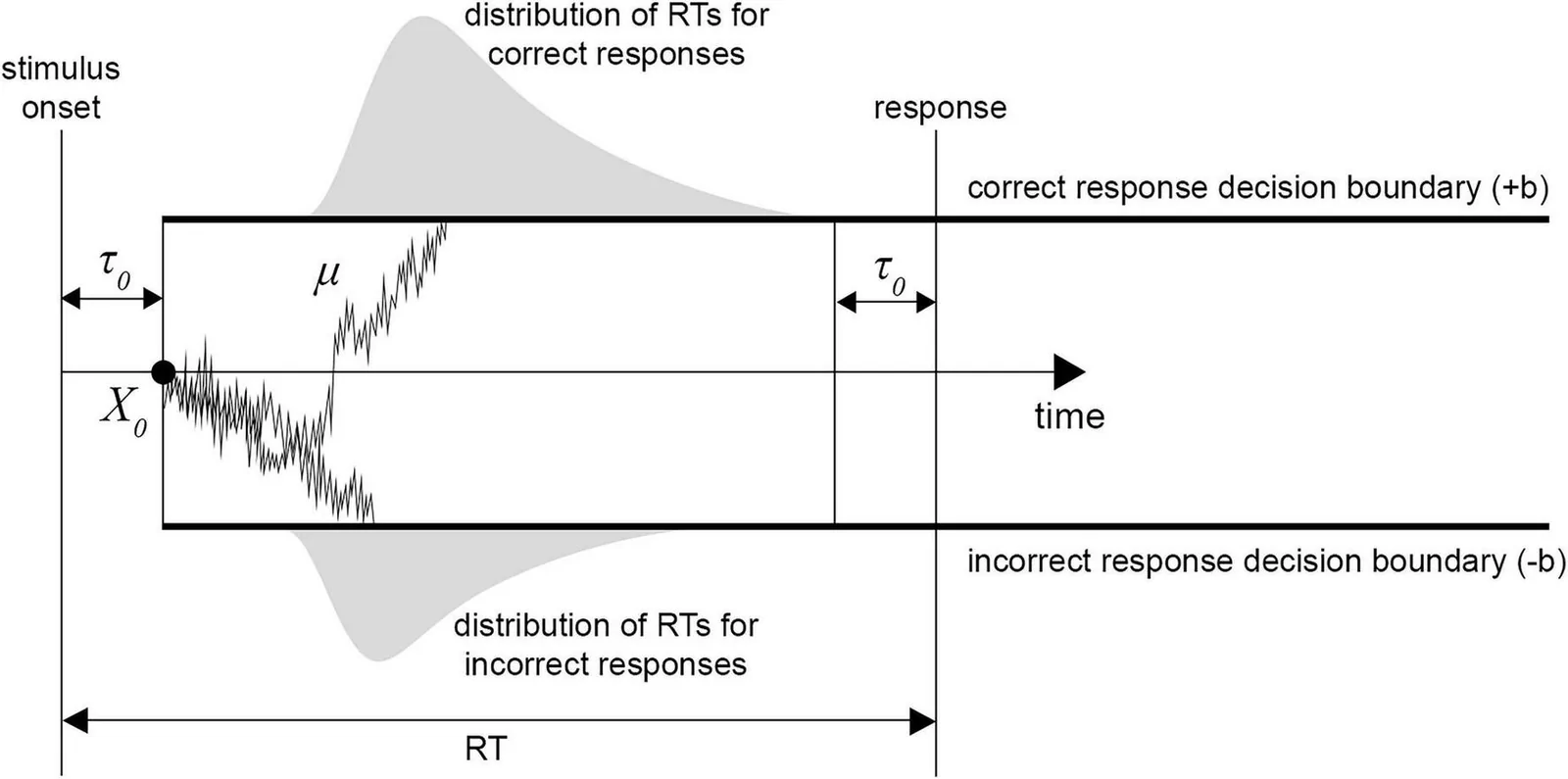
Illustration of the different drift-diffusion model (DDM) parameters. Decision-making in DDM is a noisy evidence accumulation process, denoted by gray curves. The evidence accumulation process initiates at X_0_ and evolves according to the drift rate (μ). The drift rate may differ across trials according to the Wiener process with increment dW(t) and standard deviation σ. The decision is reached when the accumulated evidence first reaches the decision boundary associated with a particular option. In a classical PDM task, the decision boundaries for correct responses and errors are set at +b and –b, respectively. Therefore, if the response is correct, V(DT) = +b, where decision time (DT) and the non-decision time (τ_0_) comprise response time (RT) together where τ_0_ encompasses information processing and motor response formation.

Second, it decomposes observed behavior into psychologically meaningful latent parameters: drift rate (μ) indexing the efficiency of evidence accumulation, boundary separation (b), indexing response caution, starting point (X_0_), showing initial bias toward one of the options, and non-decision time (τ_0_), covering stimulus encoding and motor output (see Figure 1). Third, these parameters show selective influence: task difficulty primarily affects drift rate, whereas speed-accuracy instructions primarily affect boundary separation (Voss et al., 2004). This property may allow researchers to link specific cognitive subprocesses to the activity of specific neural circuits.

While the standard DDM with four core parameters (μ, b, *X_0_*, τ_0_) is most common, several variants and extensions have been developed to address specific research questions or data constraints. These include the EZ-diffusion model (EZ-DDM) (Wagenmakers et al., 2007), a simplified version that provides closed-form equations for estimating a restricted set of parameters, useful when trial numbers are limited. For more complex designs, the Hierarchical Drift Diffusion Model (HDDM) (Wiecki et al., 2013) uses Bayesian estimation to simultaneously fit parameters at the individual and group level, improving reliability. For tasks involving more than two choices or confidence judgments, the Linear Ballistic Accumulator (LBA) model (Brown and Heathcote, 2008) is often preferred, as it assumes independent accumulators for each response alternative rather than a single diffusion process.

## 6 Age-dependent differences in evidence accumulation models

In the context of cognitive aging, applying SSMs allows researchers to move beyond age-related differences in outcomes, such as RT and accuracy, toward understanding how aging affects latent cognitive processes. Here, we review findings related to the effect of age on drift rate, boundary separation, and non-decision time, which are summarized in Supplementary Table 2.

### 6.1 Drift rate

Drift rate (μ) reflects the average speed of accumulating sensory evidence in favor of one alternative after stimulus detection. This parameter is often considered the computational analog of mental speed — that is, the baseline rate at which elementary processes of perception, analysis, and response preparation unfold (von Krause et al., 2022). In the most reviewed studies, no significant differences in drift rate were found between young and older adults across different cognitive tasks including numerosity discrimination (Ratcliff, 2008), numerical and verbal task (Kühn et al., 2011), lexical decisions task (Ratcliff et al., 2004), signal detection-like task (Ratcliff et al., 2007), and random dot motion discrimination task (Kavcic et al., 2013; McGovern et al., 2018). However, when McGovern et al. (2018) revised the DDM formulation based on neural data analysis, they observed another result. In this work, neurally informed modeling involved constraining the free parameters of DDM based on direct measurements of neural decision signals (the centro-parietal positivity and mu-beta activity) rather than relying solely on behavioral data. Interestingly, refining the classic DDM paradigm by fixing the decision boundary parameter based on neurophysiological data revealed that older adults had slower drift rates that were completely masked by conventional behavioral modeling. Decrease in drift rate in older adults were also found in brightness discrimination task (Ratcliff et al., 2007), and in face-object categorization task (Bolam et al., 2024). Some studies showed that the speed of evidence accumulation increased in older adults in the random dot motion task (Forstmann et al., 2011), and letter discrimination task (Wieschen et al., 2024). Taken together, these findings indicate that the way drift rate depends on age varies across tasks. The absence of a universal age-related decline in this parameter suggests that basic perceptual processing speed may remain relatively preserved in healthy aging.

### 6.2 Boundary separation

As SSM suggests, evidence accumulation continues until one of two preset decision thresholds (b) is reached. Crossing a threshold corresponds to the moment of decision-making and initiation of the motor response. The distance between these boundaries (boundary separation) is a key parameter reflecting response caution: wider separation implies a need for more evidence before committing to a choice, leading to higher accuracy but also longer RTs. The effect of age on boundary separation shows greater cross-task consistency than the drift rate. In the most studies, boundary separation was wider in older adults, except several studies, where no differences between age group where found (Ratcliff, 2008; Kühn et al., 2011; Kavcic et al., 2013). Greater boundary separation in older adults supports the hypothesis of age-related increases in response conservatism: a strategic tendency to accumulate more evidence before deciding, often at the expense of speed. Therefore, an increased threshold in older adults does not necessarily reflect cognitive decline. Ratcliff et al. (2006) demonstrated that with practice, thresholds in older participants could decrease significantly, approaching those of younger individuals. This finding suggests that age-related increases in conservatism may be, at least in part, an adaptive or plastic strategy rather than a fixed consequence of neural constraints.

### 6.3 Non-decision time

Non-decision time (τ_0_) is the component of SSMs unrelated to evidence accumulation. This parameter captures the combined duration of processes such as initial stimulus encoding and motor response planning and execution. In most studies reviewed, non-decision time increased with age. However, McGovern et al. (2018) observed higher values of non-decision time in young adults in the random dot motion task using the standard DDM; and Forstmann et al. (2011) did not find differences in the non-decision time in the same task. The increase in non-decision time observed in older adults aligns with the view that age-related slowing in overall RT is partly attributable to progressively longer durations of peripheral stages. Finally, practice, did not improve this parameter in older adults, suggesting that non-decision time may be relatively stable and closely tied to fundamental physiological changes (Ratcliff et al., 2006).

Taken together, computational models suggest that older adults typically adopt a more cautious strategy, reflected in a higher decision boundary, and exhibit longer non-decision times, likely reflecting sensorimotor slowing. However, drift rate, indexing the efficiency of evidence accumulation, does not show a consistent age-related decline; it may be lower, higher, or unchanged. Rather than reflecting isolated changes in individual components due to cognitive decline, Rahnev and Denison (2018) propose a framework in which age-related neurobiological changes affect all components simultaneously, leading to a shift in the overall decision process. As a result, the behavior of older adults may appear more cautious or strategically altered, not because of a single deficit, but because the decision system operates under different internal constraints. In this sense, aging can be viewed as a reconfiguration of decision processes rather than a simple decline in performance.

## 7 fMRI correlates of perceptual decision-making

In this section, we review neuroimaging studies of age-induced changes in PDM process based on functional magnetic resonance imaging (fMRI). While an extensive body of literature focuses on young adults (see Supplementary Table 3), far less work has examined differences in neural activation between young and older adults under the same tasks and instructions (see Supplementary Table 4).

### 7.1 Neural correlates of evidence accumulation

Across studies reviewed here, the fMRI literature primarily focuses on brain activity related to evidence accumulation. Regions most frequently implicated across studies include the intraparietal sulcus (IPS), anterior insula (aINS), dorsolateral prefrontal cortex (DLPFC), pre-supplementary motor area (pre-SMA), and the inferior frontal sulcus/gyrus (IFS/IFG).

The IPS has been suggested to be a homologue of the LIP region in monkeys (Grefkes and Fink, 2005), which activity is associated with evidence accumulation (Huk and Shadlen, 2005). It has been repeatedly implicated in evidence accumulation across studies (Buchsbaum et al., 2013; Ho et al., 2009; Kayser et al., 2010; Liu and Pleskac, 2011; Tosoni et al., 2008). However, some work suggests that the IPS is involved in both perceptual and motor processes (Ho et al., 2009; Liu and Pleskac, 2011; Tosoni et al., 2008). When dissociating these processes, Newton et al. (2022) found that different subregions of the IPS serve distinct functions based on their connectivity with the lateral prefrontal cortex. Specifically, a caudal subregion (cIPS), connected to the pre-premotor cortex, was sensitive to sensory coherence, whereas a rostral subregion (rIPS), connected to the dorsal premotor cortex, was involved in attentional control and motor responses.

The aINS emerges as the most consistently identified accumulator across diverse paradigms (Buchsbaum et al., 2013; Ho et al., 2009; Krueger et al., 2017; Liu and Pleskac, 2011; Morito and Murata, 2022; Ploran et al., 2007; Thielscher and Pessoa, 2007; Tremel and Wheeler, 2015). These findings suggest the aINS may serve as a hub for integrating sensory evidence, though its precise role — whether in accumulation *per se*, interoceptive signal processing related to decision confidence, or the moment of recognition — remains debated.

Neuroimaging studies of the DLPFC in PDM show considerable variability. Some studies have linked the DLPFC to evidence accumulation (Heekeren et al., 2004; Ivanoff et al., 2008). However, other studies have not consistently identified the DLPFC as an accumulator (Ho et al., 2009; Krueger et al., 2017; Liu and Pleskac, 2011), instead associating it with other functions involved in PDM, such as metacognition (Fleming et al., 2012; Vaccaro and Fleming, 2018) and conflict resolution (Van Maanen et al., 2015).

Within the lateral prefrontal cortex, other subregions—particularly the IFS and IFG—have also been identified as evidence accumulators (Buchsbaum et al., 2013; Ho et al., 2009; Liu and Pleskac, 2011; Morito and Murata, 2022; Ploran et al., 2007; Thielscher and Pessoa, 2007; Tremel and Wheeler, 2015). At the same time, the open questions remain, whether its role is content-specific, domain-general, or related to cognitive control processes.

A distinct class of accumulator regions has been identified in category-selective visual areas. Tremel and Wheeler (2015) demonstrated that the fusiform gyrus and parahippocampal gyrus exhibit accumulation-like activity patterns that are stimulus-specific: the slope of the leading edge of the BOLD response scales with reaction time for preferred stimuli but not for non-preferred stimuli. Morito and Murata (2022) classified category-selective regions—including the fusiform face area, extrastriate body area, parahippocampal place area, and lateral occipital complex—as type-C accumulators, with activity profiles modulated by stimulus preference and reflecting competition among choice alternatives. These findings indicate that evidence accumulation is not solely a property of frontoparietal regions but can also be observed in sensory areas when task demands require discrimination between competing stimulus categories.

Finally, the pre-SMA and anterior cingulate cortex (ACC) have been implicated in both evidence accumulation and the implementation of speed–accuracy trade-offs (Buchsbaum et al., 2013; Ivanoff et al., 2008; Kayser et al., 2010; Ploran et al., 2007; Thielscher and Pessoa, 2007; Van Maanen et al., 2015).

### 7.2 Neural correlates of other processes related to PDM

Regions involved in early sensory processing are consistently engaged during PDM tasks, with their activity primarily reflecting stimulus properties. In the face-object categorization task used by Heekeren et al. (2004), face-selective and house-selective regions in ventral temporal cortex represented the sensory evidence available for each stimulus category, showing stronger responses to preferred stimuli that scaled with stimulus clarity. Similarly, Ploran et al. (2007) identified sensory processors in lingual gyrus and cuneus, where activity increased gradually as stimulus information accumulated but did not show response-time-dependent shifts in peak latency. Morito and Murata (2022) further characterized sensory processors in superior, middle, and inferior occipital gyri, noting that activity in these regions peaked independently of decision timing. In the motion domain, Kayser et al. (2010), Ivanoff et al. (2008) both identified middle temporal complex (MT+) as a region where activity scaled with motion coherence but did not exhibit the temporal profile characteristic of evidence accumulation. Buchsbaum et al. (2013) confirmed that occipital regions and posterior intraparietal sulcus primarily reflected stimulus duration rather than decision-related processes, supporting the distinction between time-on-task effects and evidence accumulation.

Kim and Lee (2011) proposed that preSMA contributes to setting the decision threshold (boundary) in drift-diffusion models, thereby regulating the speed-accuracy trade-off. Impulsive perceptual choices (fast but error-prone) are thought to arise when this threshold is set too low, and IFG’s role in response inhibition (e.g., stop-signal tasks) may extend to preventing premature responses based on insufficient sensory evidence.

Several subcortical structures and the cerebellum have been implicated in PDM, though their roles are less consistently characterized (Liu and Pleskac, 2011; Thielscher and Pessoa, 2007). Ploran et al. (2007), Krueger et al. (2017) found that caudate and cerebellum showed evidence accumulation signatures. Morito and Murata (2022) classified pallidum, ventral diencephalon, and cerebellum as accumulators.

Among the subcortical structures found, the caudate nucleus, for example, is presumably involved in the accumulation of a decision variable (Ding and Gold, 2013), and the cerebellum is involved in integrating sensory information, correcting errors and preparing a motor response (Peterburs and Desmond, 2016). A comprehensive and insightful review by Jahanshahi et al. (2015) further consolidates this perspective by detailing how the fronto-striato-subthalamic-pallidal network, including the subthalamic nucleus and the caudate, sets decision thresholds and mediates the balance between evidence accumulation and impulsive action during perceptual choice.

### 7.3 Neural correlates of different components of PDM identified using SSM

In a subset of fMRI studies, SSMs have been applied directly to behavioral data to generate quantitative predictions about the BOLD signal or to interpret neural activity in terms of model parameters. Ho et al. (2009) used the LBA model to generate a quantitative prediction of the BOLD waveform: they simulated ramping neural activity, convolved it with the hemodynamic response function, and predicted a larger and temporally delayed BOLD response for harder (low-coherence) trials. They then confirmed this specific spatiotemporal pattern in the right aINS, identifying it as a supramodal accumulator. Liu and Pleskac (2011), using DDM, confirmed that stimuli coherence in random dot motion task primarily affected drift rate. They used DDM to derive a qualitative sign prediction that lower drift rate should produce higher BOLD (greater integrated activity). They tested only the main effect of coherence, with BOLD amplitude being higher for low-coherence than for high-coherence trials, and found this signature in IPS, FEF, aINS, and IFS. Kayser et al. (2010) fitted a DDM to behavioral data and confirmed that drift rate increased with coherence. They derived the prediction that slower drift (low coherence) should produce a larger integrated BOLD signal based on the assumption that the BOLD response is proportional to the integral of neural activity over the decision duration. They tested this prediction by relying primarily on the amplitude effect (low > high, i.e., higher BOLD for low-coherence trials) demonstrated via a linear trend analysis across coherence levels and found an inverse BOLD-coherence relationship in the IPS. In this area Krueger et al. (2017) used DDM to show that imposing a penalty for errors increased decision thresholds and dramatically slowed RTs; they then used up-ramp and down-ramp general linear model regressors to dissociate ramping (accumulator) from boxcar activity, identifying bilateral aINS, cerebellum, and caudate as candidate accumulation regions. Tremel and Wheeler (2015) used HDDM to fit behavioral data and directly regressed trial-wise BOLD slope measures from face- and house-selective regions in the inferior temporal cortex onto drift rate, showing that the slope of the rising BOLD response correlated positively with drift rate.

In conclusion, SSMs serve a dual role in fMRI studies of PDM. At a theoretical level, they provide a principled framework for interpreting BOLD signal dynamics in terms of evidence accumulation, enabling researchers to distinguish decision-related regions from those involved in sensory or attentional processes. At a quantitative level, fitting these models to behavioral data allows estimation of latent parameters such as drift rate, which can then be used to generate testable predictions about the magnitude and timing of the BOLD response. However, direct regression of model parameters onto fMRI activity remains relatively rare.

### 7.4 Age-related differences in neurocorrelates of perceptual decision-making

Our literature search reveals a noticeable scarcity of studies examining the neurocorrelates of PDM in older adults. This is likely due to several factors. First, the BOLD signal depends not only on neural activity but also on vascular health, which declines with age (De Vis et al., 2015; West et al., 2019; Wright and Wise, 2018; Wu et al., 2023), making fMRI signals more difficult to analyze and interpret. Second, cerebral atrophy complicates spatial normalization, as standard templates derived from young adults fit older brains poorly (Rorden et al., 2012). Third, age-related sensory deterioration (Dully et al., 2018) further complicates the study of PDM.

Despite these challenges, recent studies have identified age-related differences in fMRI signals relevant to PDM. Older adults exhibit reduced BOLD signal variability, which is associated with decreased cognitive efficiency (Armbruster-Genç et al., 2016). This reduction is particularly in default mode and fronto-parietal regions (Kumral et al., 2020) and is accompanied by diminished modulation of variability when transitioning from rest to task (Garrett et al., 2013). Habak et al. (2019) in Gabor patches task showed that ACC activity in older adults increased with task difficulty, whereas no such relationship was observed in younger adults, suggesting that the processing of difficulty changes with age. Similarly, Mayhew and Kourtzi (2013) in Glass pattern stimuli found distinct patterns of neural activation: younger adults engaged a broad network spanning occipitotemporal, parietal, and frontal regions, whereas older adults showed changes primarily in parietal areas, with no clear evidence of frontal recruitment. In contrast, Seider et al. (2021) reported that increasing age was associated with greater activity in frontal (premotor and middle frontal gyri) and temporal (superior temporal gyri) regions, and no regions showed age-related decreases in activation in match-to-sample task. Here, increased activity was also observed in the right primary somatosensory cortex, suggesting that even early sensory regions may require additional recruitment in older adults. Together, these findings indicate that older adults may rely on more effortful processing—particularly in frontal regions associated with top-down attentional control—to maintain performance levels comparable to younger adults (Reuter-Lorenz and Cappell, 2008).

Additionally, overactivation of the frontoparietal network has been observed in older adults (Hsieh et al., 2025; Rieck et al., 2017), along with impaired functional interactions with the default mode network. Reduced suppression of activity in this network is considered a marker of neurocognitive aging (Turner and Spreng, 2015). However, the relationship between this hyperactivation and task performance remains controversial, with some evidence suggesting that it may not be purely neural in origin but partly reflects age-related vascular changes, such as altered neurovascular coupling and cerebrovascular reactivity (Stiernman et al., 2023). Furthermore, a meta-analysis by Spreng et al. (2010), encompassing over 80 studies, reported that older adults exhibit greater prefrontal activation during cognitive tasks and reduced occipital activation during perceptual tasks—a pattern consistent with compensatory or dedifferentiation accounts of aging.

### 7.5 Differences in association between model parameters and neurocorrelates between young and old adults

Only few works connect neuroimaging with modeling to understand differences between young and older adults. Kühn et al. (2011) provide evidence directly linking computational model parameters to brain activity in older adults. By correlating DDM parameters with task-related BOLD signals in numerical and verbal task, they found that key brain–behavior relationships were preserved with age. Specifically, striatal activity remained associated with response caution (decision threshold), and activity in the IPL continued to track drift rate (evidence accumulation efficiency) in both young and older adults. Although older adults differed in their overall behavior the mapping between brain activity and decision components did not change. This suggests that aging may alter the values of decision parameters, but not the underlying neural architecture linking these parameters to specific brain regions.

Forstmann et al. (2011) examined age-related slowing in PDM by combining the LBA model with diffusion-weighted imaging (DWI) in random dot motion task. They found that older adults adopt higher response thresholds—indicating greater caution—and adjust them less flexibly under speed pressure compared to younger adults. Structurally, younger adults showed stronger white matter connectivity between the pre-SMA and the striatum, and stronger connectivity was associated with more flexible threshold adjustment. These results suggest that older adults’ reluctance to adopt faster, riskier responses may reflect structural limitations, with age-related degradation of cortico-striatal pathways impairing the neural mechanisms that support rapid adjustment of decision thresholds.

## 8 Causal mapping of PDM with non-invasive brain stimulation

Non-invasive brain stimulation (NIBS) offers an opportunity to move beyond correlational neuroimaging studies by enabling the testing of causal relationships between activity in specific cortical regions and behavioral outcomes (Polanía et al., 2018). In addition to advancing our understanding of fundamental brain functions, NIBS also has important therapeutic implications, particularly in older adults. For example, a number of meta-analyses and review papers have examined the use of NIBS in older populations for the treatment of depression (Palm et al., 2016; Yang Y. et al., 2025), Alzheimer’s disease (Elder and Taylor, 2014; Li et al., 2024), mild cognitive impairment (Chou et al., 2020; Saleh et al., 2023), Parkinson’s disease (Guo et al., 2020; Zhang et al., 2022), and post-stroke conditions (Elsner et al., 2017; Klomjai et al., 2015). Moreover, NIBS has been widely applied to study various cognitive functions in older adults (for a review, see Martins et al., 2016).

In studies of cognitive functions in older adults TMS and transcranial direct current stimulation (tDCS) are commonly used. They are differ fundamentally in their mechanisms. TMS uses an alternating magnetic field to induce electrical currents that directly trigger action potentials in cortical neurons, offering high spatial and temporal precision for causal mapping of specific brain regions (spatial resolution approximately 25 mm^2^, or about 5 mm accuracy for distinguishing excitable vs. non-excitable cortical areas, temporal resolution about 1 ms) (Priori et al., 2009), but it requires expensive equipment and carries a risk of seizure (Rossi et al., 2021). Nevertheless, Iriarte and George (2018) explicitly state that TMS demonstrates good tolerability in older patients. In contrast, tDCS delivers a weak constant current that only modulates neuronal excitability, providing lower spatial resolution (typical electrode size ∼2,500 mm^2^, which is approximately 100 times larger than the focus of TMS) (Priori et al., 2009) but greater portability, safety, and ease of use for multi-day protocols (Bikson et al., 2016).

In the context of studying PDM and its age-induced changes, NIBS provides a powerful tool for gaining a mechanistic understanding of age-related cognitive deficits across different stages of the decision process. A key methodological advance in recent years has been the integration of neuromodulatory interventions with mathematical models of decision-making. When combined with SSMs, this approach allows researchers to move beyond identifying whether a brain region is involved in decision-making, and instead determine which specific component of the process—such as drift rate, boundary separation, or non-decision time—is affected.

According to our literature review, most NIBS studies on PDM have been conducted exclusively in younger adults. This creates a critical gap in the literature: we do not yet know whether the causal roles established for various brain regions in younger adults hold in the aging brain, where structural and functional reorganization may fundamentally alter how these regions contribute to decision processes. This section reviews NIBS studies that have combined neuromodulation with SSMs, with particular attention to the single study that has directly compared younger and older adults (see Supplementary Table 5).

Transcranial magnetic stimulation, particularly can induce lasting changes in cortical excitability beyond the stimulation period, making it well-suited for establishing causal contributions of specific brain regions to PDM. In cognitive neuroscience research, two main classes of TMS protocols are commonly used to study PDM. The first is low-frequency (≤1 Hz) repetitive TMS (rTMS), which typically decreases cortical excitability of the targeted region for a period lasting from minutes to up to an hour after stimulation (Chen et al., 1997). The second is theta-burst stimulation (TBS), a patterned rTMS protocol (three impulses at 50 Hz, repeated every 200 ms) that mimics endogenous oscillatory activity and is both more efficient and more potent than conventional rTMS (Huang et al., 2005). TBS has two variants with opposing effects. Continuous TBS (cTBS) is an uninterrupted 20 or 40-s train of pulses (300 or 600 pulses) which induces long-lasting suppression of cortical excitability thought to involve long-term depression (LTD)-like mechanisms, while intermittent TBS (iTBS) delivered in a 2-s on, 8-s off pattern for 190 s (600 pulses) produces facilitation of cortical excitability via long-term potentiation (LTP)-like mechanisms (Huang et al., 2005; Lowe et al., 2018). Both cTBS and iTBS have been successfully applied to study PDM in young adults.

In articles reviewed, the DLPFC or pre-SMA are most often chosen as areas for stimulation. The DLPFC was chosen based on neuroimaging studies showing its involvement in evidence accumulation (Heekeren et al., 2004, 2006) and speed-accuracy tradeoff (van Veen et al., 2008). The pre-SMA was targeted due to its proposed role in setting the decision threshold via projections to the striatum, as formalized in the striatal theory of the speed-accuracy tradeoff (Bogacz et al., 2010; Forstmann et al., 2008, 2011).

Several studies have established links between the modulation of DLPFC activity and the efficiency of evidence accumulation. In a key study, Philiastides et al. (2011) applied low-frequency (1 Hz) rTMS for 12 min to the left DLPFC to transiently inhibit neural activity. Following stimulation, participants showed decreased accuracy, increased RT, and, critically, a significant reduction in drift rate as estimated by the DDM in face-object discrimination task. These effects were specific to the first two blocks of the testing session (about 10 min), consistent with the temporary nature of the rTMS after-effects. This finding provided evidence that the left DLPFC is involved in the rate of sensory evidence integration. Similarly, Wittkuhn et al. (2018) used 1 Hz rTMS over the left DLPFC during a sequential decision-making task and found that stimulation impaired performance accuracy and reduced drift rate, particularly in a delayed rewards condition that required integrating information across multiple decision states. Notably, using a cross-over design, the authors showed that inhibitory rTMS in the first session impaired the initial acquisition of the task’s sequential structure, and this deficit persisted into a second session, even without further stimulation, highlighting the role of the left DLPFC in learning complex contingencies.

While the DLPFC is linked to the efficiency of evidence accumulation, the pre-SMA has been consistently implicated in the cautiousness of the decision policy — the decision threshold. Tosun et al. (2017) applied continuous inhibitory cTBS to the right pre-SMA in random dot motion task. While mean RT and accuracy were unchanged, DDM analysis revealed a robust and selective increase in boundary separation and drift rate. Berkay et al. (2018) extended these findings using a bidirectional TBS protocol in random dot motion task. They confirmed that inhibitory cTBS over the pre-SMA increased the decision threshold, while excitatory iTBS over the same region decreased the threshold. This bidirectional effect provides compelling evidence that the pre-SMA is not merely correlated with, but directly controls, the setting of the response criterion, consistent with the striatal theory of the speed-accuracy tradeoff (Forstmann et al., 2011).

A more refined functional organization along the rostrocaudal axis of the frontal cortex was demonstrated by Rahnev et al. (2016) in Gabor patches task. Using cTBS over three progressively rostral frontal regions — the frontal eye fields (FEF), the DLPFC, and the anterior prefrontal cortex (aPFC) — they showed a double dissociation. Inhibiting the caudal FEF disrupted the ability to select an attended stimulus, reflected in a reduced RT advantage for attended vs. unattended targets. Inhibiting the mid-lateral DLPFC impaired the ability to adjust the decision criterion under speed-accuracy instructions, reflected in a smaller RT difference between speed and accuracy trials. Finally, inhibiting the rostral aPFC unexpectedly improved metacognitive sensitivity (the correspondence between confidence and accuracy), suggesting a role for this region in evaluating decisions. These results suggest a hierarchical organization of PDM in the lateral frontal cortex.

It is also worth noting that not all NIBS methods used in SSM-based PDM studies produce a neuromodulatory effect measurable at the model parameters level. For example, the studies reviewed here that used tDCS in young adults did not observe a significant effect on DDM parameters. Hollander et al. (2016) conducted three independent experiments testing the effect of anodal tDCS over the pre-SMA on the speed-accuracy trade-off in random dot motion task. Using DDM to analyze performance, they found no effect of tDCS on any parameter, including boundary separation. Similarly, Turkakin et al. (2018) attempted to replicate findings that tDCS over the primary motor cortex (M1) could bias hand choice. They found no effect on choice behavior or DDM parameters.

When interpreting findings from NIBS studies, it is crucial to distinguish between the strength of causal inference afforded by different experimental designs. For instance, bidirectional TMS protocols that combine both inhibitory and excitatory stimulation of the same brain region provide substantially stronger evidence for a causal role than single-session, unidirectional rTMS protocols, which can only demonstrate that a region is necessary under specific task conditions. Moreover, several well-controlled tDCS studies have failed to modulate DDM parameters, highlighting the heterogeneity of the causal evidence in NIBS research. Therefore, causal claims derived from NIBS should be interpreted as region-, task-, and protocol-specific rather than as evidence of generalizable neural mechanisms underlying perceptual decision-making.

The existing evidence obtained using NIBS regarding the potential contribution of cortical regions to PDM remains limited. Although a modest body of work exists in younger adults, such studies are very scarce in older adults. To the best of our knowledge there is only one study that directly compares these two age groups using NIBS with PDM task (Fujiyama et al., 2022). In this study, anodal tDCS over the right rIFG or pre-SMA were used while participants performed a flashing grid task. The results revealed a clear age-dependent effect of neuromodulation. For decision-making speed (Go RT; i.e., response time to the choice stimulus when no stop signal was presented), anodal tDCS over both rIFG and pre-SMA sped up responses in young adults. However, in older adults, faster Go RTs were observed only following pre-SMA stimulation; rIFG stimulation had no effect. A similar age-by-site interaction was found for inhibitory control (stop-signal reaction time). In young adults, response inhibition improved only after rIFG stimulation, whereas in older adults, it improved only after pre-SMA stimulation. Importantly, while this study provides critical age-comparative behavioral data, it did not formally estimate DDM parameters. Thus, it remains unknown whether the observed behavioral improvements were driven by changes in drift rate, decision threshold, or non-decision time — a key question for future research.

## 9 Conclusion

The present review demonstrates that the integration of mathematical modeling, neuroimaging, and neuromodulation provides a powerful framework for decomposing PDM into its constituent cognitive and neural components. Sequential sampling models, particularly the DDM, offer a principled approach to disentangling latent processes such as evidence accumulation, response caution, and non-decision time, thereby overcoming the limitations of traditional behavioral measures such as reaction time and accuracy. When combined with neuroimaging and NIBS, these models enable a more precise mapping between cognitive processes and their underlying neural substrates.

A limitation of the present review is that it exclusively considered experimental tasks employing visual stimuli. Given the well-documented age-related decline in sensory function, it is reasonable to expect that similar age-related effects on behavioral and model parameters would also be observed across other sensory modalities. This expectation is supported by several studies employing auditory and tactile stimuli (Pitts and Sarter, 2018; Yang X. et al., 2025).

A central conclusion of this review is that, despite substantial progress in understanding the architecture of PDM in younger adults, research on the aging brain remains markedly limited. Existing evidence suggests that aging is associated with a systematic reconfiguration of the decision process rather than a uniform decline. Older adults typically exhibit increased response caution and prolonged non-decision times, while age-related effects on drift rate remain inconsistent across tasks. At the neural level, aging is characterized by structural degradation, altered neurochemical dynamics, and changes in large-scale network organization, which together may fundamentally alter how decision-related regions operate and interact.

Non-invasive brain stimulation studies in young adults have provided important preliminary insights into the neural basis of perceptual decision-making (PDM), suggesting that regions such as the dorsolateral prefrontal cortex (DLPFC) contribute to evidence accumulation, whereas the pre-supplementary motor area (pre-SMA) is involved in setting decision thresholds. However, the strength of the causal evidence remains heterogeneous, and the findings have not been consistently replicated across studies. Thus, although NIBS provides a powerful tool for investigating causal relationships, the current evidence is best viewed as offering local, region-, task-, and protocol-specific mechanistic insights rather than a comprehensive understanding of the neural processes underlying PDM.

Moreover, it remains unclear whether these functional mappings generalize to older adults. The limited available evidence suggests that while the relationship between model parameters and neural activity may be preserved, the effectiveness of neuromodulation differs across age groups, likely reflecting age-related changes in brain structure and connectivity. This gap highlights an important direction for future research. There is a need for studies that systematically combine computational modeling, neuroimaging, and neuromodulation within aging populations. Such integrative approaches would allow researchers to determine not only whether specific brain regions contribute to decision-making in older adults, but also how age-related changes modify their functional roles and responsiveness to stimulation. Moreover, incorporating SSMs into NIBS studies in older adults would provide critical insights into which components of the decision process can be selectively targeted to mitigate cognitive decline.

From a translational perspective, this line of research holds significant promise. A mechanistic understanding of how aging affects distinct components of decision-making could inform the development of targeted neuromodulatory interventions aimed at improving cognitive performance in older adults. Ultimately, bridging the gap between computational models, brain mechanisms, and causal interventions represents a key step toward a more comprehensive and clinically relevant understanding of cognitive aging.
