# Supplementary material for: Integrating computational modeling, neuroimaging, and neuromodulation to decompose perceptual decision-making in healthy aging: a review of methods, findings, and gaps
Source: Front Aging Neurosci. 2026 Aug 7;18:1906284. doi: 10.3389/fnagi.2026.1906284 (PMC13493598; doi:10.3389/fnagi.2026.1906284)
Supplement: Supplementary file 1 [file Data_Sheet_1.docx]

Supplementary Material

1 Supplementary Tables

Supplementary Table 1. Brief description of the PDM tasks used in the reviewed studies

| **Task** | **Description** |
| --- | --- |
| Brightness discrimination | subject need to decide decides whether the figure presented on the screen was light or dark (Ratcliff et al., 2006, 2007). A 64×64 pixel square was represented on the screen, consisting of black and white dots followed by a mask. Stimulus duration 50, 100, and 150 ms. Levels of brightness (value for the probability of a pixel being white): .350, .425, .475, .525, .575, and .650. |
| Gradual contrast-change detection task | subject observe the flickering checkerboard pattern and should notice a gradual decrease in contrast (McGovern et al., 2018) |
| Face-object discrimination | subject need to decide whether the masked in noise image shows a human face or another object (e.g., a car, house, or tool) |
| Face emotion discrimination | subject need to decide whether a presented face was either fearful or disgusted (Habak et al., 2019) |
| Flanker task | five horizontal arrows are displayed on the screen. Subject need to ignore the side arrows (flankers) and indicate the direction of the center arrow (left or right) (Revie & Metzler-Baddeley, 2024) |
| Flashing grid task | subject viewed a dynamically shuffling 20×20 grid of blue and orange cells with a fixed proportion of the dominant color, made a speeded perceptual decision about which color was more frequent by pressing a corresponding key, and attempted to inhibit their response on a subset of trials when a red stop signal appeared (Fujiyama et al., 2022) |
| Gabor patches | subject need to detect or identify the orientation (e.g., vertical, horizontal, or tilted) of a striped grating pattern (Habak et al., 2019) |
| Glass patterns | subject need to perceive a global form (e.g., concentric circles or radial rays) made of randomly positioned dot pairs with a specific correlation (Mayhew and Kourtzi, 2013) |
| Letter discrimination | subject need to decide whether letter was presented on the screen and then masked (Ratcliff et al., 2007) |
| Letter-number-discrimination task | subject need to decide whether it was letter or number presented on the screen. Numbers from “1” to “9” and letters from “A” to “I” served as stimuli and were presented one at a time (Wieschen et al., 2024) |
| Lexical decision | subject need to identify whether it was a word or a nonword demonstrated on the screen. *Pseudoword test* – from each word, a pronounceable pseudoword was generated by randomly replacing all of the vowels with other vowels (Ratcliff et al., 2004, 2007). *Letter strings test* – pool of random unpronounceable letter strings, created by randomly sampling letters from the alphabet and then by removing those strings that were pronounceable were demonstrated. High-, low-, and very low-frequency words were used (Ratcliff et al., 2004, 2007). |
| Masked Letter Discrimination | subject need to whether one of two letters presented on the screen and then masked. Stimulus durations: 10, 20, 30, 40, 50, and 60 ms (Ratcliff et al., 2006, 2007). |
| Match-to-sample task | subject need to determine which of the two lower stimuli is identical to the upper one according to a given characteristic (spatial location, shape, velocity), and press the corresponding button (Seider et al., 2021) |
| Noise-occluded pictures unmasking | subject need to identify a degraded or partially hidden object while noise is progressively reduced (Ploran et al., 2007) |
| Numerical task | subject need to determine whether the number presented is even or odd. Stimulus masking 50 ms after demonstration (Kühn et al., 2011) |
| Numerosity discrimination task | subject need to determine there were more or less than 50 points in a 10×10 array (Ratcliff, 2008) |
| Random dot-motion task | subject need to determine the direction of moving dots embedded in dynamic visual noise. *Continuous version* – there is no clear division into trials, goals arise in a stream of noise (McGovern et al., 2018) |
| Selecting more squares of the same color | subjects need to judge whether black or white squares were more frequent in a 20×20 grid and responded within 800 ms by pressing a key with their left or right hand (Turkakin et al., 2018) |
| Signal detection-like task | subject need to judge whether the distance between two dots was large or small. Manipulation of stimulus difficulty by varying the distances between the two dots (Ratcliff et al., 2007) |
| Verbal task | subject need to determine whether the letter is a vowel or a consonant. Stimulus masking 50 ms after demonstration (Kühn et al., 2011) |
| 3-stage deterministic Markov decision task | task requiring two-alternative forced choices at each state to learn optimal action sequences that maximized cumulative rewards, with immediate versus delayed reward conditions manipulating the need to integrate cross-state action-outcome contingencies (Wittkuhn et al., 2018) |

**Supplementary Table 2.** Overview of studies examining age effects on behavioral measures and DDM parameters in PDM

| Article | Aim | Subjects (y.o.) | Task, modality | Paradigm | RT | Accuracy | Sequential-sampling model | Model parameters | | |
| --- | --- | --- | --- | --- | --- | --- | --- | --- | --- | --- |
|  |  |  |  |  |  |  |  | μ | b | $\tau_{0}$ |
| Ratcliff, 2008 | To identify components of processing  that differ between young and older participants | 19 Y  (20.8 ± 1.3)  19 O  (69.2 ± 4.5) | *Numerosity discrimination task* | Alternating "speed" and "accuracy" blocks. The standard RT task – free response time; response-signal task – to respond within 300 ms | Y < O | Y < O | DDM | ns | Y < O  In response signal – ns | Y < O |
| Kühn et al., 2011 | To identify neural correlates of evidence accumulation | 24 Y (20-31)  15 O (65-80) | *Numerical task* | Mask duration depended on age | Y < O | Y > O | DDM | ns | ns | Y < O |
|  |  |  | *Verbal task* |  |  |  |  |  |  |  |
| McGovern et al., 2018 | To investigate the impact of ageing on the neural decision process | 35 Y (m.a. 22)  31 O (m.a. 74) | *RDM (continuous)* | Free choice | Y < O | Y > O | DDM and neurally-informed DDM (niDDM) | ns in DDM  Y > O in niDDM | Y < O in DDM | Y > O in DDM |
|  |  | 38 Y (m.a. 22)  38 O (m.a. 74) | *Gradual contrast-change detection task* |  | ns | Y < O |  | Y < O in DDM  ns in niDDM | Y < O in DDM  ns in niDDM | ns |
| Ratcliff et al., 2004 | To compare lexical-decision performance for older and younger adults | 54 Y (m.a. 19.8)  44 O (m.a. 68.5) | *Lexical Decision (words and pseudoword)* | Free choice | Y < O | Y < O | DDM | ns | Y < O | Y < O |
|  |  | 54 Y (m.a. 67.2)  40 O (m.a. 20.2) | *Lexical Decision (words and random letter strings)* |  |  |  |  |  |  |  |
| Ratcliff et al., 2006 | To evaluate the impact of practice on changing behavioral characteristic and model parameters | 27 Y (m.a. 20.9)  27 O (m.a. 69.3) | *Letter Discrimination* | Alternating blocks for "speed" and "accuracy" | Y < O  O – strong effect of practice | Y > O  O – strong effect of practice | DDM | Y > O  O - strong effect of practice | Y < O  O – strong effect of practice | Y < O  no impact of practice |
|  |  | 26 Y (m.a. 19.9)  24 O (m.a. 68.8) | *Brightness Discrimination* |  |  | Y > O in “accuracy” |  | ns |  |  |
| Bolam et al., 2024 | Assessment of age-related changes in multisensory perception | 212 subjects  (43.5 ± 18,5)* | *Face-object categorization* | Respond as quickly and accurately as possible | Y < O | Y > O | HDDM | Y > O | Y < O  in high coherence | Y < O  only in low coherence |
| Ratcliff et al., 2007 | Comparison of the results of older adults aged 75-90 years with previously obtained data for 60-75 years and young people | Y (students, from previous studies)  O1 (60-75)  O2 (75-90) | *Signal detection-like task* | Alternating blocks for "speed" and "accuracy", except for lexical decision tasks | “speed”: Y < O1 < O2  “accuracy”: Y < O1, O2 | ns | DDM | ns | Y < O1  for “speed” O1 < O2 | Y < O1  O1 < O2 |
|  |  |  | *Letter discrimination* |  | Y < O1 < O2 | Y > O1, O2  for stimuli with high spatial frequency |  | Y > O1 | Y < O1, O2 | Y < O1 |
|  |  |  | *Brightness discrimination* |  | “speed”:  Y < O1 < O2  “accuracy”:  Y < O2  O1 < O2 | Y, O1 > O2  for stimuli with low spatial frequency |  | O1 > O2 | O1 < O2 | Y < O1 |
|  |  |  | *Lexical decision (words and pseudowords)* |  | Y < O1 < O2 | Y < O1, O2 |  | ns | Y < O1, O2 | Y < O1 |
|  |  |  | *Lexical decision random (words and letter strings)* |  | Y < O1 < O2 | Y < O1, O2 |  | ns | Y < O1, O2 | Y < O1  O1 < O2 |
| Forstmann et al., 2011 | To investigate the neuroanatomical basis of age-related slowing of PDM | 12 Y (25.3 ± 3.5)  12 O (64.5 ± 5) | *RDM* | Instructions: “faster”, “more precise”, “neutral” | Y < O | Y > O | LBA | Y < O | Y < O  in O decrease is smaller in “faster” | ns |
| Wieschen et al., 2024 | To identify qualitative differences in evidence accumulation | 40 Y (18-35)  40 O (63-80) | *Letter-number-discrimination task* | no manipulation of noise level, coherence, or duration of the stimulus | Y < O | Y < O | DDM | for letters:  Y < O | Y < O | Y < O |
| Revie and Metzler-Baddeley, 2024 | To study mechanisms underlying age-related slowing of RT | 25 Y (18-29)  25 O (62 – 80) | *flanker task* | Congruent and incongruent conditions | Y < O | ns | EZ-DDM | ns | Y < O | Y < O |
| Kavcic et al., 2013 | To study the influence of age on EEG correlates of motion perception (P1, N1) | 12 Y (21-37)  19 O (58-89) | *RDM* | Difficulty control: 1) all dots move coherently; 2) the direction of each dot was randomized within a range of 320° relative to the average direction (left or right) | Y < O | ns | EZ-DDM | ns | ns | Y < O |

v – drift rate, a – boundary separation, Ter – non-decision time, Y – young adults, O – older adults, ns – no significance, m.a. – middle age. ">" indicates a greater mean RT, greater number of correct answers or greater model parameter (drift rater, boundary separation, non-decision time) value in an age group. The definition applies to all tasks and all conditions unless otherwise stated.

* Age was analyzed as a continuous variable.

**Supplementary Table 3**. fMRI studies of perceptual decision-making in young adults

| Article | Subjects | Sequential sampling model | Task | Result |
| --- | --- | --- | --- | --- |
| Heekeren et al., 2004 | 12 Y (m.a. 31.1) | X | Face-object discrimination | *Greater response in perithreshold stimuli*:  FEF (BA 6), SEF, IPS  *Greater response in suprathreshold stimuli:*  SFS in DLPFC (BA 8/9), PCC (BA1), SFG (BA9)  *Higher-level decision-making areas, uses output from face- and house-selective regions to form a decision:*  SFS in L DLPFC (BA8/9) |
| Thielscher and Pessoa, 2007 | 25 Y (m.a. 23 ± 3) | X | Face emotion discrimination | *Task-related activation (100% faces vs baseline)*:  FG, STS, amygdala, aINS, putamen, IPS, ACC, IFG, Thal  *Perceptual choice (classification of neutral face)*:  *For fearful faces:* CF, STS, SPG, AI, ACC, MFG, IFG, OFC, Thal.  *For disgust faces*: putamen, R aINS  *Stimulus-related activations (100% fearful vs 100% disgust)*:  *Fearful*: L LG, L FG, R EC, aINS, R IFS, R OG, R Thal, L caudate  *Disgust*: R MOG, L IPS, R MTG  *Decision making (RT and fMRI amplitude)*:  ACC, L MFG, IFG/AI |
| Buchsbaum et al., 2013 | 5 Y (22-38) | X | RDM (time constraint that  varied on a trial-by-trial basis) | *Time on task (correlation with stimulus duration):*  Occ, MT+, posterior IPS  *Decision-related processing (correlation with motion coherence):*  aINS, preSMA, IFS, mIPS |
| Ho et al., 2009 | 12 Y (age information not provided) | LBA | RDM (red and blue dots, 4AFC) | *Larger and temporally extended response on hard vs easy trials:*  R Ins, IPS, FEF, MFC, R IFG, R SFG, R MFG  *Modality-dependent evidence accumulation:*  *Saccades*: IPS  *Manual*: L central sulcus  *Supramodal*: aINS |
| Kayser et al., 2009 | 6 Y (23-37) | rate-diffusion model | RDM | *Higher activity in hard trials:*  IPS, MT+, FEFs, MFG, ACC/SMA, aINS  *Evidence accumulation*:  IPS |
| Krueger et al., 2017 | 20 Y (m.a. 22.7) | DDM | RDM (with SAT) + control | *Evidence accumulation candidates (RDM > control, parameter estimates greater for the up-ramp vs down-ramp regressor):*  MOG (BA 19), aINS, L cerebellum, R caudate, L ITG (BA 37), FG  *Greater activation during RDM vs control (general processes):*  MOG (BA 19), FEF, MFG, PMC, SPL + IPL (BA 7), caudate + red nucleus, P premotor, aINS, cerebellum |
| Ploran et al., 2007 | 25 Y (19–29) | as theoretical core | Noise-occluded pictures unmasking | *Sensory processors:*  LG, cuneus  *Evidence accumulation*:  L IPS, MFG, cerebellum, IFG, FG, IOG, SOG,  *Moment of recognition*:  caudate, IPL, Thal, IFG, R DAC, aINS/fO, R PG, R MFG, L MFG/pre-SMA |
| Liu and Pleskac, 2011 | 9 Y (m.a. 27) | DDM | RDM (with manipulation of the response mode, foreknowledge of response mode) | *Modality dependent activity:*  *Saccades:* IPS (1-4), FEF, vPrCS, SEF, MT+  *Manual*: PMd, M1, PoC, SMA  *Evidence accumulation (higher fMRI*  *response on low coherence trials):*  IPS (IPS1-3), FEF, vPrCS, IFS, aINS |
| Ivanoff et al., 2008 | 13 Y (20-31) | as theoretical core | RDM (continuous version with SAT) | *Sensory processing:*  MT+  *Evidence accumulation:*  pLPFC, pre-SMA |
| Tosoni et al., 2008 | 12 Y (m.a. 24.2) | X | Face-object discrimination | *Modality-dependent:*  *Saccades: p*IPS, FEF  *Manual:* precuneus, aPRR, pPRR, SMC, FRR  *Evidence accumulation (*:  aPRR, pPRR, pIPS  *Main effect of sensory evidence, modality independent (BOLD greater in hard trials):*  PFC, INS, preSMA, anterior cingulate |
| Tremel and Wheeler, 2015 | 16 Y (21-26) | HDDM | Face-object discrimination | *Stimulus-specific:*  *Faces discrimination:*  IFG  *Object discrimination*:  parahippocampal gyrus  *Non-specific*:  *Accumulators*: FG, IOG, IFG  *Moment of recognition*: aINS, Thal |
| Morito and Murata, 2022 | 14 Y (m.a. 26.8) | as theoretical core | Human-scene discrimination | *Sensory processors:*  SOG, MOG, IOG, OFuG, R ITG, L Occ  *Accumulators:*  *Type A - activity peaks matching the decision timing:*  MFG, PrG, OpIFG, MSFG, SeMC, fO, aINS, OrIFG, TrIFG, pallidum, ventral DC, cerebellum  *Type B - activity peaks after decision:*  R MFG, R PG, SPL, L AG, L ITG, FG, cerebellum  *Type C – content-specific:*  fusiform face area, extrastriate body area, parahippocampal place area, lateral occipital complex |

Abbreviations: m.a. – mean age; BA – Brodmann area; 4AFC – four-alternative forced-choice task; symbol “/” – near; Y – younger adults, O – older adults, a – anterior, p – posterior, Op – opercular part, Or – orbital part, Tr – triangular part, d – dorsal, vm – ventromedial, RDM – random dot-motion task, SAT – speed-accuracy tradeoff.

Areas: aINS - anterior insula, aC – anterior cingulate, ACC – anterior cingulate cortex, AG - angular gyrus, CF – calcarine fissure, DAC – dorsal anterior cingulate, DC – diencephalon, DIPs - dorsal intraparietal sulcus, DLPFC – dorsolateral prefrontal cortex, EC – entorhinal cortex, FEF – frontal eye field, FG – fusiform gyrus, fO – frontal operculum, FRR – frontal reach region, IFG – inferior frontal gyrus, IFJ – inferior frontal gyrus, IFS – inferior frontal sulcus, INS – insula, IOG – inferior occipital gyri, IPL – inferior parietal lobule, IPS – intraparietal sulcus, IT – Inferior temporal cortex, ITG - inferior temporal gyrus, LG – lingual gyrus, LO – lateral occipital area, LOC – lateral occipital cortex, M1 – primary motor cortex, MFC – medial frontal cortex, MFG – middle frontal gyrus, MOG – middle occipital gyri, MSFG – superior frontal gyrus medial segment, Occ – occipital pole, OFC – orbitofrontal cortex, OFuG – occipital fusiform gyrus, OG – orbital gyrus, OPS - occipital parietal sulcus, PCC – posterior cingulate cortex, PG – precentral gyrus, pLPFC – posterior lateral prefrontal cortex, PMC – premotor cortex, PMd – dorsal premotor area, PMv – ventral premotor area, POC – posterior occipital regions, POIPs - parieto-occipital intraparietal sulcus, PPL – posterior parietal lobes, preSMA – presupplementary motor area, PRR – parietal reach region, SEF – supplementary eye field, SeMC – sensory motor cortex, SFG – superior frontal gyrus, SFS - superior frontal sulcus, SMA – supplementary motor area, SMC – supplementary motor cortex, SMG – supramarginal gyrus, SOG – superior occipital gyrus, SPG – superior parietal gyrus, SPL – superior parietal lobule, STG – superior temporal gyrus, STS – superior temporal sulcus, Thal – thalamus, TPJ – temporal parietal junction, VIPs - ventral intraparietal sulcus, VLPFC – ventrolateral prefrontal cortex, vPrCS - ventral portion

**Supplementary Table 4**. fMRI studies of perceptual decision-making in older adults

| Article | Subjects | Model | Task | Result |
| --- | --- | --- | --- | --- |
| Kühn et al., 2011 | 24 Y (20-31)  15 O (65-80) | DDM | Numerical and verbal task  Pretest and posttest – before and after 100-days training | *More activity in pretest (transition from effort-dependent task execution to more automatic):*  dACC, aINS, L IFJ, L IFG, R FG  *More activity in posttest (default mode network activity for automatic task execution):*  vmPFC, precuneus, STG  *Correlation with DDM parameters (greater activity – higher parameter value)*:  *Drift-rate:* IPL  *Threshold*: putamen  *Non-decision time*: L SMG  No age-related changes |
| Forstmann et al., 2011 | 12 Y (22-32)  12 O (61-79) | LBA | RDM (with SAT + feedback) | Y had stronger connections from right pre-SMA to the right striatum. Significant negative correlation between tract strength and response caution. |
| Habak et al., 2019 | 20 Y (24-32)  8 O (62-82) | X | Gabor patches | In O, activity in ACC increased with increasing task difficulty (i.e., when moving from easy to difficult discriminations), whereas in young adults there was no such relationship. |
| Mayhew and Kourtzi, 2013 | 10 Y (m.a. 21)  10 O (m.a. 71)  +  control 8 Y (m.a. 22) and 8 O (m.a. 70) | X | Glass patterns  Control (orthogonal letter detection task) | *fMRI activation patterns that relate to behavioral improvement after training*:  *Young adults*: LO, VIPs, POIPs, DIPs, OPS, PostCS, PMv, PMd, PCC, MFG, IFG  *Older adults*: VIPs, POIPs, DIPs, OPS, PostCS, ACC  In older adults visual shape learning engages primarily parietal regions. After training the slopes of the fMR-metric functions were steeper in young than older participants in occipitotemporal. |
| Seider et al., 2021 | 40 O (51-91) | X | Match-to-sample task | *Positively correlated with age (greater age, greater activity)*:  *Spatial arrangement*: R S1  *Shape*: R PMC, MFG  *Movement*: MFG, STG |

Abbreviations: m.a. – mean age; Y – younger adults, O – older adults, R or L – right or left hemisphere, a – anterior, d – dorsal, vm – ventromedial, SAT – speed-accuracy tradeoff.

Areas: ACC – anterior cingulate cortex, DIPs - dorsal intraparietal sulcus, FG – fusiform gyrus, IFG – inferior frontal gyrus, IFJ – inferior frontal gyrus, INS – insula, IPL – inferior parietal lobule, LO – lateral occipital area, MFG – middle frontal gyrus, OPS - occipital parietal sulcus, PCC – posterior cingulate cortex, PFC – prefrontal cortex, PMd – dorsal premotor area, PMv – ventral premotor area, POIPs - parieto-occipital intraparietal sulcus, PostCS – post-central sulcus, pre-SMA – presupplementary motor area, S1 – primary somatosensory cortex, SMG – supramarginal gyrus, STG – superior temporal gyrus, VIPs - ventral intraparietal sulcus.

**Supplementary Table 5**. Studies of perceptual decision-making with NIBS

| Article | Subjects (y.o.) | Task | Stimulation area | Model | Type of stimulation | Stimulation parameters | Results |
| --- | --- | --- | --- | --- | --- | --- | --- |
| Berkay et al., 2018 | 17 Y (21-29) | RDM | preSMA | HDDM | TBS: cTBS, iTBS. Control: vertex inhibitory/excitatory stimulation | 3 imp. at 50 Hz, rep. 200 ms,  80% AMT.  cTBS - 40 s (600 imp.)  iTBS - 2 s imp., 8 s pause, 190 s (600 imp.) | Threshold increases with inhibitory stimulation and decreases with excitatory stimulation |
| Georgiev et al., 2016 | 15 Y (m.a.30) | RDM | R DLPFC, preSMA | DDM | cTBS | 3 imp. 20 ms, rep. 200 ms, 40 s (600 imp.), 80% AMT | - TMS in the pre-SMA reduced boundary separation;  - Only at stimuli with high coherences drift rate decreases with TMS in R DLPFC |
| Philiastides et al., 2011 | 11 Y (23-29) | Face-object discrimination | L DLPFC | DDM | rTMS | 1 Hz, 12 min, 720 stimuli, 110% RMT | Immediately after TMS (first 2 blocks), accuracy decreased, RT increased, and drift rate decreased |
| Tosun et al. 2017 | 24 Y (19-24) | RDM | preSMA | HDDM | cTBS | 3 imp. at 50 Hz, rep. 200 ms, 40 s (600 imp.), 80% AMT | - Threshold increased;  - Drift rate increased;  - RT and accuracy unchanged |
| Rahnev et al., 2016 | 17 Y (21-30) | Gabor patches | FEF, DLPFC, aPFC | two-accumulator decision model with confidence based on evidence difference | cTBS | 5 bursts of 3 imp. at 50 Hz, 40 s (600 imp.), 80% RMT | - FEF: decreased RT difference between attended and unattended stimuli;  - DLPFC: decreased RT difference between the "speed" and "accuracy" conditions;  - aPFC: increased consistency between confidence and accuracy of responses |
| Wittkuhn et al., 2018 | 31 Y (19-32) | 3-stage deterministic Markov decision task | L DLPFC | EZ-DDM | rTMS | 1 Hz, 20 min., 100% RMT | - Stimulation only in the first session worsened the accuracy of choosing the optimal sequence of actions, but only in the delayed reward condition;  - RT increased;  - Drift speed decreased; |
| (Hollander et al., 2016) | 44 Y (m.a. 23.8) | RDM | Anode above FCz according to the 10-20 system | DDM | tDCS | 1 mA, 13 min, linear fade-in/fade-out of 15 s | No significant effect of anodal tDCS on any of the DDM parameters |
| Luber et al., 2020 | 13 Y (m.a. 24.6) | Face-object discrimination | R и L LOC | Spatiotemporal model of three distributed brain networks that activate in temporal sequence to enable visual decision-making | ppTMS | 50 ms pause, 100% RMT, SOA: −200,200,400,450,500 ms | TMS at 400 ms significantly increased RT compared to control (500 ms) |
| Turkakin et al., 2018 | 24 Y (m.a. 20.3) | Selecting more squares of the same color | M1 (anode above С3, cathode above С4) | Bayesian estimation of DDM | tDCS | 1.5 mA, 15 min, fade-in/fade-out of 10 s | - tDCS did not result in a shift in hand choice or RT;  - DDM parameters did not changed |
| Fujiyama et al., 2022 | 42 Y (18-34)  41 O (60-80) | flashing grid task, stop signal task | IFG | DDM as theoretical core | tDCS | 1.5 mA, 20 min | RT in young adults decreased with stimulation of both areas, but in older adults, reaction speed improved only with preSMA stimulation |

imp. – impulse, rep. – repeat, AMT – active motor threshold, RMT – resting motor threshold, LOC - lateral occipital cortex, SOA - stimulus onset asynchronies, TBS – theta-burs TMS, cTBS - continuous TBS, iTBS – intermittent TSB, rTMS – repetitive transcranial magnetic stimulation, ppTMS – paired-pulse TMS, tRNS – transcranial random-noise stimulation

**Reference list:**

Berkay, D., Eser, H. Y., Sack, A. T., Çakmak, Y. Ö., & Balcı, F. (2018). The modulatory role of pre-SMA in speed-accuracy tradeoff: A bi-directional TMS study. *Neuropsychologia*, *109*, 255–261. https://doi.org/10.1016/j.neuropsychologia.2017.12.031

Bolam, J., Diaz, J. A., Andrews, M., Coats, R. O., Philiastides, M. G., Astill, S. L., & Delis, I. (2024). A drift diffusion model analysis of age-related impact on multisensory decision-making processes. *Scientific Reports*, *14*(1), 14895. https://doi.org/10.1038/s41598-024-65549-5

Buchsbaum, B. R., Erickson, D. T., & Kayser, A. S. (2013). Decomposing Effects of Time on Task Reveals an Anteroposterior Gradient of Perceptual Decision Regions. *PLOS ONE*, *8*(8), e72074. https://doi.org/10.1371/journal.pone.0072074

Forstmann, B. U., Tittgemeyer, M., Wagenmakers, E.-J., Derrfuss, J., Imperati, D., & Brown, S. (2011). The Speed-Accuracy Tradeoff in the Elderly Brain: A Structural Model-Based Approach. *Journal of Neuroscience*, *31*(47), 17242–17249. https://doi.org/10.1523/JNEUROSCI.0309-11.2011

Fujiyama, H., Tan, J., Puri, R., & Hinder, M. R. (2022). Influence of tDCS over right inferior frontal gyrus and pre-supplementary motor area on perceptual decision-making and response inhibition: A healthy ageing perspective. *Neurobiology of Aging*, *109*, 11–21. https://doi.org/10.1016/j.neurobiolaging.2021.09.014

Georgiev, D., Rocchi, L., Tocco, P., Speekenbrink, M., Rothwell, J. C., & Jahanshahi, M. (2016). Continuous Theta Burst Stimulation Over the Dorsolateral Prefrontal Cortex and the Pre-SMA Alter Drift Rate and Response Thresholds Respectively During Perceptual Decision-Making. *Brain Stimulation*, *9*(4), 601–608. https://doi.org/10.1016/j.brs.2016.04.004

Habak, C., Seghier, M. L., Brûlé, J., Fahim, M. A., & Monchi, O. (2019). Age Affects How Task Difficulty and Complexity Modulate Perceptual Decision-Making. *Frontiers in Aging Neuroscience*, *11*. https://doi.org/10.3389/fnagi.2019.00028

Heekeren, H. R., Marrett, S., Bandettini, P. A., & Ungerleider, L. G. (2004). A general mechanism for perceptual decision-making in the human brain. *Nature*, *431*(7010), 859–862. https://doi.org/10.1038/nature02966

Ho, T. C., Brown, S., & Serences, J. T. (2009). Domain General Mechanisms of Perceptual Decision Making in Human Cortex. *Journal of Neuroscience*, *29*(27), 8675–8687. https://doi.org/10.1523/JNEUROSCI.5984-08.2009

Hollander, G. de, Labruna, L., Sellaro, R., Trutti, A., Colzato, L. S., Ratcliff, R., Ivry, R. B., & Forstmann, B. U. (2016). Transcranial Direct Current Stimulation Does Not Influence the Speed–Accuracy Tradeoff in Perceptual Decision-making: Evidence from Three Independent Studies. *Journal of Cognitive Neuroscience*, *28*(9), 1283–1294.

Ivanoff, J., Branning, P., & Marois, R. (2008). fMRI Evidence for a Dual Process Account of the Speed-Accuracy Tradeoff in Decision-Making. *PLOS ONE*, *3*(7), e2635. https://doi.org/10.1371/journal.pone.0002635

Kavcic, V., Martin, T., & Zalar, B. (2013). Aging effects on visual evoked potentials (VEPs) for motion direction discrimination. *International Journal of Psychophysiology*, *89*(1), 78–87. https://doi.org/10.1016/j.ijpsycho.2013.05.012

Kayser, A. S., Buchsbaum, B. R., Erickson, D. T., & D’Esposito, M. (2009). *The Functional Anatomy of a Perceptual Decision in the Human Brain*. *103*(3), 1179–1194. https://doi.org/10.1152/jn.00364.2009

Krueger, P. M., van Vugt, M. K., Simen, P., Nystrom, L., Holmes, P., & Cohen, J. D. (2017). Evidence accumulation detected in BOLD signal using slow perceptual decision making. *Journal of Neuroscience Methods*, *281*, 21–32. https://doi.org/10.1016/j.jneumeth.2017.01.012

Kühn, S., Schmiedek, F., Schott, B., Ratcliff, R., Heinze, H.-J., Düzel, E., Lindenberger, U., & Lövden, M. (2011). Brain Areas Consistently Linked to Individual Differences in Perceptual Decision-making in Younger as well as Older Adults before and after Training. *Journal of Cognitive Neuroscience*, *23*(9), 2147–2158. https://doi.org/10.1162/jocn.2010.21564

Liu, T., & Pleskac, T. J. (2011). Neural correlates of evidence accumulation in a perceptual decision task. *Journal of Neurophysiology*, *106*(5), 2383–2398. https://doi.org/10.1152/jn.00413.2011

Luber, B., Jangraw, D. C., Appelbaum, G., Harrison, A., Hilbig, S., Beynel, L., Jones, T., Sajda, P., & Lisanby, S. H. (2020). Using Transcranial Magnetic Stimulation to Test a Network Model of Perceptual Decision Making in the Human Brain. *Frontiers in Human Neuroscience*, *14*. https://doi.org/10.3389/fnhum.2020.00004

Mayhew, S. D., & Kourtzi, Z. (2013). Dissociable circuits for visual shape learning in the young and aging human brain. *Frontiers in Human Neuroscience*, *7*. https://doi.org/10.3389/fnhum.2013.00075

McGovern, D. P., Hayes, A., Kelly, S. P., & O’Connell, R. G. (2018). *Reconciling age-related changes in behavioural and neural indices of human perceptual decision-making*. *2*(12), 955–966.

Morito, Y., & Murata, T. (2022). Accumulation System: Distributed Neural Substrates of Perceptual Decision Making Revealed by fMRI Deconvolution. *The Journal of Neuroscience*, *42*(24), 4891–4912. https://doi.org/10.1523/JNEUROSCI.1062-21.2022

Philiastides, M. G., Auksztulewicz, R., Heekeren, H. R., & Blankenburg, F. (2011). Causal Role of Dorsolateral Prefrontal Cortex in Human Perceptual Decision Making. *Current Biology*, *21*(11), 980–983. https://doi.org/10.1016/j.cub.2011.04.034

Ploran, E. J., Nelson, S. M., Velanova, K., Donaldson, D. I., Petersen, S. E., & Wheeler, M. E. (2007). Evidence Accumulation and the Moment of Recognition: Dissociating Perceptual Recognition Processes Using fMRI. *Journal of Neuroscience*, *27*(44), 11912–11924. https://doi.org/10.1523/JNEUROSCI.3522-07.2007

Rahnev, D., Nee, D. E., Riddle, J., Larson, A. S., & D’Esposito, M. (2016). Causal evidence for frontal cortex organization for perceptual decision making. *Proceedings of the National Academy of Sciences*, *113*(21), 6059–6064. https://doi.org/10.1073/pnas.1522551113

Ratcliff, R. (2008). Modeling aging effects on two-choice tasks: Response signal and response time data. *Psychology and Aging*, *23*(4), 900–916. https://doi.org/10.1037/a0013930

Ratcliff, R., Thapar, A., Gomez, P., & McKoon, G. (2004). A Diffusion Model Analysis of the Effects of Aging in the Lexical-Decision Task. *Psychology and Aging*, *19*(2), 278–289. https://doi.org/10.1037/0882-7974.19.2.278

Ratcliff, R., Thapar, A., & McKoon, G. (2006). Aging, practice, and perceptual tasks: A diffusion model analysis. *Psychology and Aging*, *21*(2), 353–371. https://doi.org/10.1037/0882-7974.21.2.353

Ratcliff, R., Thapar, A., & McKoon, G. (2007). Application of the diffusion model to two-choice tasks for adults 75-90 years old. *Psychology and Aging*, *22*(1), 56–66. https://doi.org/10.1037/0882-7974.22.1.56

Revie, L., & Metzler-Baddeley, C. (2024). Age-related fornix decline predicts conservative response strategy-based slowing in perceptual decision-making. *Aging Brain*, *5*, 100106. https://doi.org/10.1016/j.nbas.2024.100106

Seider, T. R., Porges, E. C., Woods, A. J., & Cohen, R. A. (2021). An fMRI study of age-associated changes in basic visual discrimination. *Brain Imaging and Behavior*, *15*(2), 917–929. https://doi.org/10.1007/s11682-020-00301-x

Thielscher, A., & Pessoa, L. (2007). *Neural Correlates of Perceptual Choice and Decision Making during Fear–Disgust Discrimination*. *27*(11), 2908–2917.

Tosoni, A., Galati, G., Romani, G. L., & Corbetta, M. (2008). Sensory-motor mechanisms in human parietal cortex underlie arbitrary visual decisions. *Nature Neuroscience*, *11*(12), 1446–1453. https://doi.org/10.1038/nn.2221

Tremel, J. J., & Wheeler, M. E. (2015). Content-specific evidence accumulation in inferior temporal cortex during perceptual decision-making. *NeuroImage*, *109*, 35–49. https://doi.org/10.1016/j.neuroimage.2014.12.072

Turkakin, E., Akbıyık, S., Akyol, B., Gürdere, C., Çakmak, Y. Ö., & Balcı, F. (2018). Differential Bilateral Primary Motor Cortex tDCS Fails to Modulate Choice Bias and Readiness in Perceptual Decision Making. *Frontiers in Neuroscience*, *12*. https://doi.org/10.3389/fnins.2018.00410

Wieschen, E. M., Makani, A., Radev, S. T., Voss, A., & Spaniol, J. (2024). Age-Related Differences in Decision-Making: Evidence Accumulation is More Gradual in Older Age. *Experimental Aging Research*, *50*(5), 537–549. https://doi.org/10.1080/0361073X.2023.2241333

Wittkuhn, L., Eppinger, B., Bartsch, L. M., Thurm, F., Korb, F. M., & Li, S.-C. (2018). Repetitive transcranial magnetic stimulation over dorsolateral prefrontal cortex modulates value-based learning during sequential decision-making. *NeuroImage*, *167*, 384–395. https://doi.org/10.1016/j.neuroimage.2017.11.057
